# Comparative Biomechanical Analysis of Kirschner Wire Fixation in Dorsally Displaced Distal Radius Fractures

**DOI:** 10.3390/life14121684

**Published:** 2024-12-19

**Authors:** Awad Dmour, Ștefan-Lucian Toma, Alin-Marian Cazac, Stefan Dragos Tirnovanu, Nicoleta Dima, Bianca-Ana Dmour, Dragos Cristian Popescu, Ovidiu Alexa

**Affiliations:** 1 Department of Orthopedics and Traumatology, Faculty of Medicine, “Grigore T. Popa” University of Medicine and Pharmacy, 700115 Iasi, Romania; awad.dmour@d.umfiasi.ro (A.D.); stefan-dragos.tirnovanu@d.umfiasi.ro (S.D.T.); ovdiu.alexa@umfiasi.ro (O.A.); 2 Materials Science Department, Materials Science and Engineering Faculty, Gheorghe Asachi Technical University of Iasi, 700050 Iasi, Romania; alin-marian.cazac@academic.tuiasi.ro; 3 Department of Orthopaedics and Traumatology, “Sf. Spiridon” Emergency Universitary Hospital, 700115 Iasi, Romania; 4 Department of Internal Medicine, “Grigore T. Popa” University of Medicine and Pharmacy, 700115 Iași, Romania; nicoleta.dima@umfiasi.ro (N.D.); bianca-ana.gherasim-dmour@d.umfiasi.ro (B.-A.D.); 5 Department of III Internal Medicine Clinic, “St. Spiridon” County Clinical Emergency Hospital, 700111 Iași, Romania

**Keywords:** distal radius fractures, biomechanical comparison, intra-focal technique, Kapandji, interfragmentary technique, turkey tarsometatarsus model

## Abstract

Objective: This study aims to evaluate and compare the biomechanical performance of two Kirschner (K) wire configurations—the intra-focal and interfragmentary techniques—for the fixation of dorsally displaced distal radius fractures. The study also assesses the impact of K-wire diameter (1.6 mm vs. 2.0 mm) on mechanical stability. Methods: Sixty fresh turkey tarsometatarsus bones were selected and divided into four groups based on the K-wire configuration and diameter used. Fractures were created at standardized locations, and each bone was stabilized using either the intra-focal also known as modified Kapandji (Ka) or interfragmentary technique. Mechanical testing, including axial compression and flexion tests, was performed to assess the biomechanical stability of each configuration. Results: The interfragmentary configuration consistently demonstrated superior biomechanical performance compared to the intra-focal technique. Specifically, the use of 2.0 mm K-wires resulted in significantly higher axial stiffness (13.28 MPa) and load at break (3070 N) compared to the 1.5 mm wires. Confidence intervals further supported the robustness of these findings. The interfragmentary technique, especially with thicker K-wires, provided greater load-bearing capacity and stiffness. Conclusion: The interfragmentary technique with 2.0 mm K-wires offers superior mechanical stability compared to the intra-focal technique, making it the preferred choice for stabilizing comminuted extra-articular distal radius fractures. These findings suggest that adopting this technique may reduce the risk of postoperative complications such as fracture displacement or malunion. Further research involving osteoporotic bone models and clinical trials is recommended to validate these findings in real-world settings.

## 1. Introduction

Distal radius fractures (DRFs) are the most common upper limb fractures, particularly prevalent in the elderly, with global incidence continuing to rise [1]. These fractures present a significant burden on healthcare systems [2] due to their complexity and potential complications, such as malunion, stiffness, and long-term disability, which can significantly affect patients’ quality of life. Additionally, a significant percentage of DRFs lead to distal radioulnar joint (DRUJ) instability and symptomatic wrist pain [3,4]. Effective treatment methods are crucial for minimizing these risks and reducing healthcare costs.

**Figure 1:**
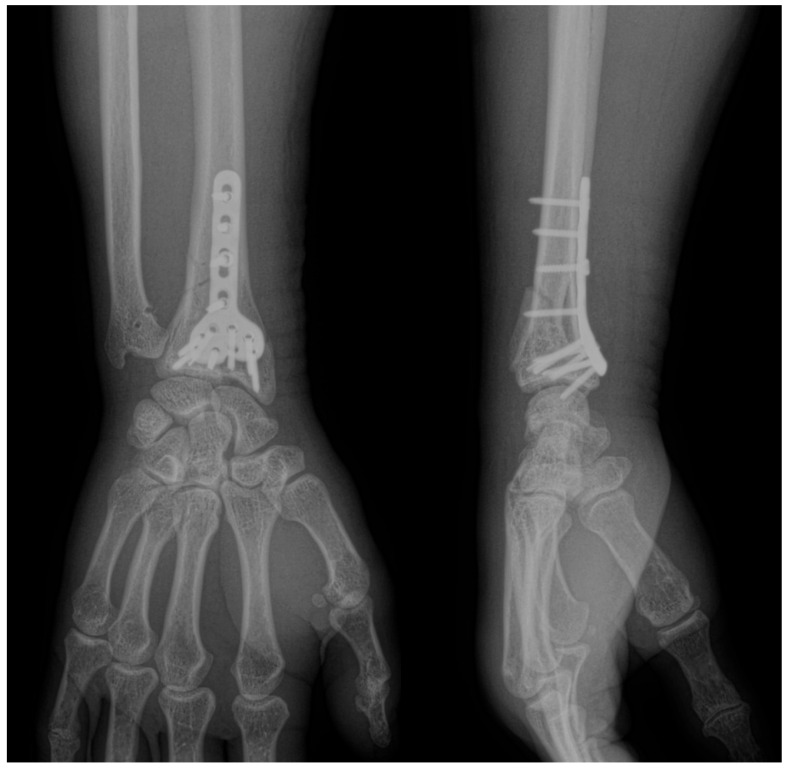
Anteroposterior and lateral views of distal radius fractures fixed with a volar locking plate.

**Figure 2:**
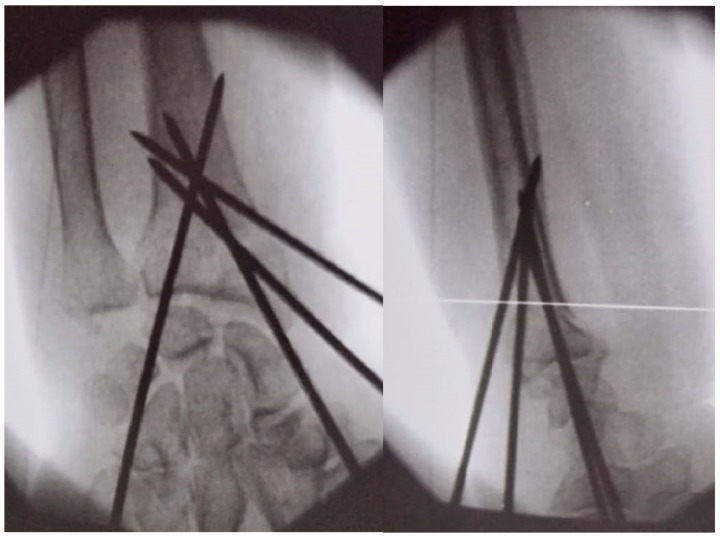
Anteroposterior and lateral intraoperative fluoroscopy views of dorsally displaced distal radius fracture (DDDRF) fixed with different sizes of k-wires and a modified Kapandji method.

Two primary surgical techniques are commonly used to manage distal radius fractures (DRFs): open reduction with volar plates (Figure 1) and percutaneous fixation using Kirschner wires (K-wires) (Figure 2) [5]. While volar plates are frequently adopted for their advantages [6], K-wire fixation remains a viable alternative due to its reduced procedure duration and lower associated costs. However, there is ongoing debate regarding the optimal number, size, and configuration of K-wires necessary for effective fracture stabilization, with the biomechanical properties of various arrangements largely underexplored [5,7].

Two common K-wire techniques are the interfragmentary method (Willenegger and Guggenbuhl technique) and the intra-focal technique also known as the modified Kapandji (Ka) technique. The interfragmentary method places oblique K-wires crossing the fracture and anchoring in the opposite cortex [8]. The intra-focal technique uses K-wires perpendicular to the fracture site, angled into the opposite cortex to prevent dorsal translation [9]. Modifications, like the Fritz-modified Kapandji, add stability with a third wire [10]. However, consensus on the ideal number, size, and configuration remains unclear [11].

Research demonstrates that the stability of K-wire constructs can vary significantly depending on the technique employed. A recent study found that the interfragmentary technique offers improved biomechanical stability [12]. However, there is a notable lack of direct biomechanical comparisons between different K-wire configurations and sizes. While the meta-analyses of clinical trials indicate that volar plates may yield superior early postoperative outcomes, such as enhanced grip strength and range of motion, K-wire fixation remains a preferred choice due to its cost-effectiveness and efficiency [7,13,14].

To address this research gap, we conducted a biomechanical analysis comparing two commonly used K-wire configurations—the intra-focal and interfragmentary methods—using a turkey tarsometatarsus bone model. This model serves as a reliable and cost-effective analog for the human distal radius, providing a reproducible platform for evaluating fracture fixation methods. By testing various wire configurations under controlled axial compression and flexion forces, this study aims to offer valuable insights into the optimal K-wire setup for enhancing mechanical stability in dorsally displaced distal radius fractures (DDDRFs).

The objective of this study is to compare the biomechanical performance of two common K-wire configurations—the three-wire intra-focal technique and the three-wire interfragmentary technique—in the fixation of simulated distal radius fractures. Additionally, we aim to evaluate the impact of wire diameter (1.6 mm vs. 2.0 mm) on mechanical stability. We hypothesize that the interfragmentary technique, particularly with larger K-wires, will demonstrate superior axial stiffness and load-bearing capacity compared to the intra-focal technique.

## 2. Materials and Methods

### 2.1. Specimen Collection and Preparation

**Figure 3:**
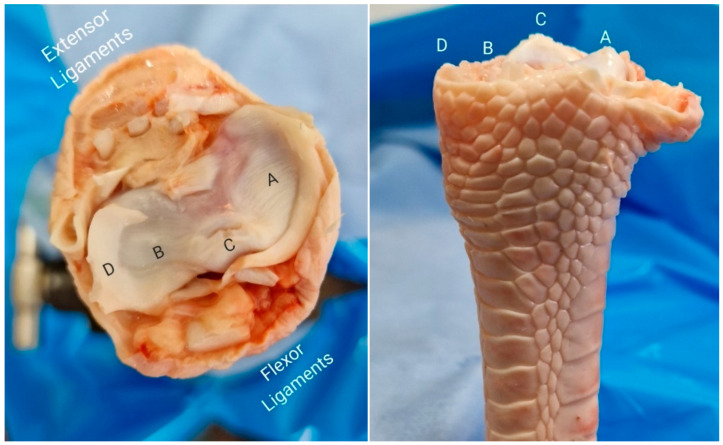
Turkey tarsometatarsus anteroposterior and distal articular surface, used as a model for distal radius: (A) Scaphoid fossa. (B) Lunate fossa. (C) Lister’s tubercle. (D) Radial styloid process.

A total of 60 fresh turkey tarsometatarsus bones (turkey shank bones) of similar age were selected for this biomechanical study due to their structural similarities to the human distal radius (Figure 3), making them suitable analogs for testing fixation techniques. Turkey bones, being readily available bones, have been frequently utilized as effective models for human bone biomechanics, offering a practical and reliable option for evaluating surgical procedures [12,15,16].

Each bone’s length and midshaft diameter were measured using a digital caliper with an accuracy of ±0.01 mm. These measurements were used for the normalization of biomechanical data and to ensure consistency across all the specimens. Bones showing any signs of pre-existing damage or structural abnormalities were excluded from the study. This ensured consistency in factors like bone density [14].

The specimens were divided into four experimental groups (n = 15 per group), each representing a combination of K-wire configuration (intra-focal or interfragmentary) and K-wire diameter (1.6 mm or 2.0 mm).

### 2.2. Fracture Creation

**Figure 4:**
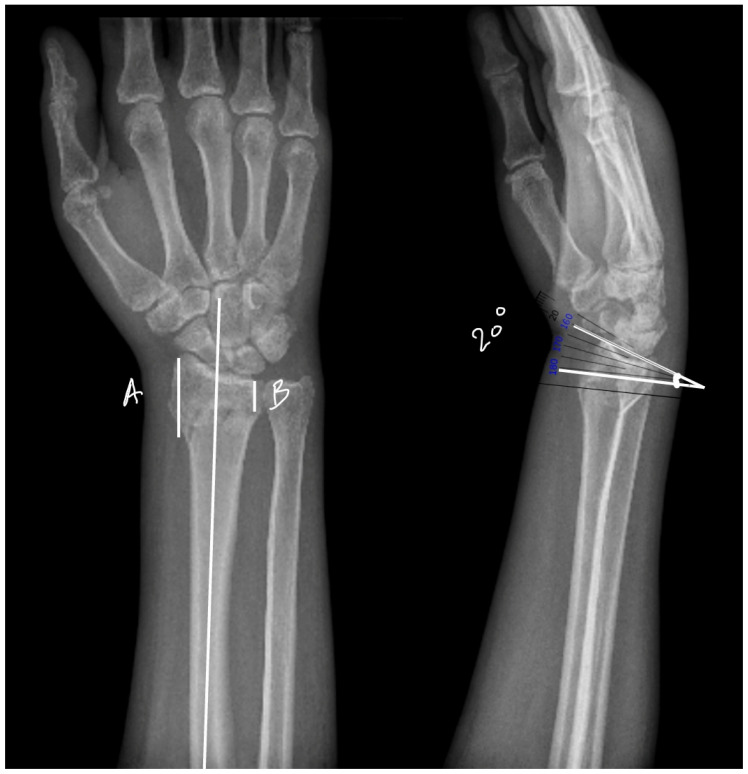
Dorsally displaced distal radius fracture measurements on anteroposterior (AP) and lateral radiographs. On AP views, we can calculate (A) lateral side measurement of the fracture line distance from the articular surface, and (B) medial side measurement of the fracture line distance from the articular surface. And on lateral views, we can calculate the mean angle between the radiocarpal joint line and fracture line.

To ensure consistency and clinical relevance in the experimental model, preoperative radiographs of patients with extra-articular distal radius fractures were obtained from Romania, Iasi County emergency hospital “Sfântul Spiridon”. The X-rays were analyzed to determine the key anatomical parameters of the distal radius. In the anterior–posterior (AP) view, the distance from the articular surface to the fracture line was measured from both the medial and lateral borders of the radius with a mean between the two of 23.4 mm (±3.2 mm) (Figure 4). This measurement was used as a reference to guide the positioning of the fracture in the turkey bones.

Lateral radiographs were also used to measure the angle of the fracture relative to the radiocarpal joint, with a mean fracture angle of 19.7 degrees (±8.9 degrees). Using this information, a hand-held surgical saw was used to create fractures at an angle of approximately 20 degrees to the long axis of the bone, with the cuts positioned 20 mm from the distal articular surface, replicating typical clinical fracture patterns. Each K-wire was controlled to penetrate 60–70% of the cortical bone, leaving part of the bone structure intact to maintain stability during biomechanical testing.

**Figure 5:**
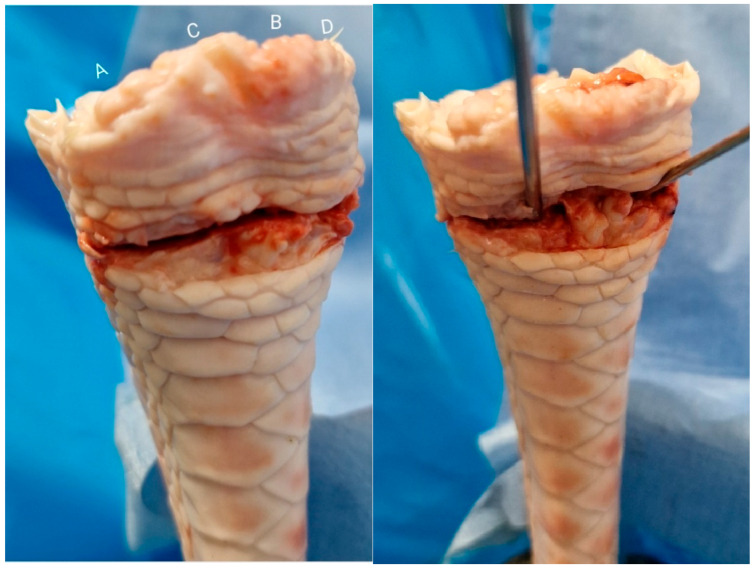
Turkey bone preparation with dorsal cuts and the insertion of K-wires. (A) Scaphoid fossa. (B) Lunate fossa. (C) Lister’s tubercle. (D) Radial styloid process.

During the fracture creation process, the bones were stabilized using a standardized fixation device, which securely held the specimens to ensure uniformity in the angle and depth of the cuts. This setup minimized variability between specimens, ensuring that the fractures were consistent across all the samples. Measurements of each bone’s length and midshaft diameter were recorded prior to fracture creation to facilitate biomechanical calculations and ensure accurate comparisons (Figure 5). Associated soft tissue injuries, such as those involving the TFCC or radioulnar ligaments, are common during distal radius fractures and play a critical role in fracture stability [17,18]. That is why we aimed to preserve as much soft tissue as possible within the turkey bone models while making the cuts, and used surgical blades for the soft tissues and sharp saws for the bone cuts.

### 2.3. K-Wire Fixation

Following the introduction of fractures, each bone was stabilized using either the three-wire intra-focal technique or the three-wire interfragmentary technique. K-wires were inserted with a drill to ensure consistent insertion pressure and depth.

**Figure 6:**
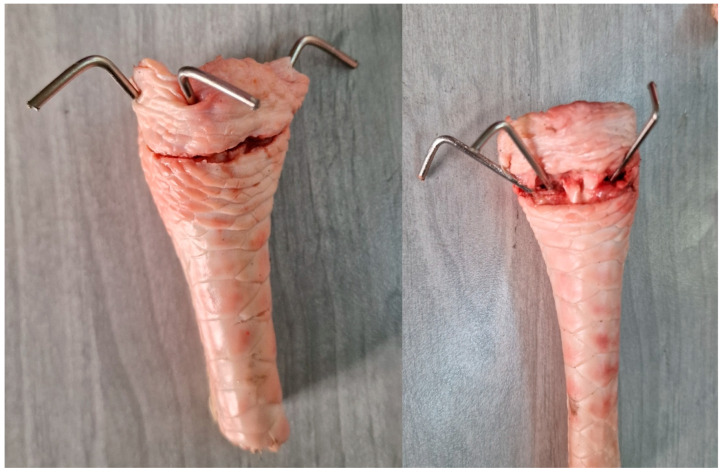
Turkey bones prepared with interfragmentary technique using 2.0 mm K-wire and intra-focal technique using 1.6 mm K-wire.

For the intra-focal technique, the K-wires were inserted intra-focally, perpendicular to the fracture site, and then angled obliquely to engage the opposite cortex [9,19]. This configuration served as a buttress to prevent dorsal displacement of the fracture. In the interfragmentary technique, one K-wire was inserted through the radial styloid and two additional wires from the dorsal aspect of the bone [10,20]. All the wires were inserted to a depth sufficient to anchor them within the bone without protruding through the opposite side (Figure 6).

Precise wire intervals were maintained and a custom fixture secured the bones to prevent movement during insertion. To minimize variability, all the fixations were performed by the same surgeon. Studies highlight that accurate hardware placement enhances stability, while poor placement can compromise fixation, especially in osteoporotic bone [14,21].

### 2.4. Mechanical Testing Setup

**Figure 7:**
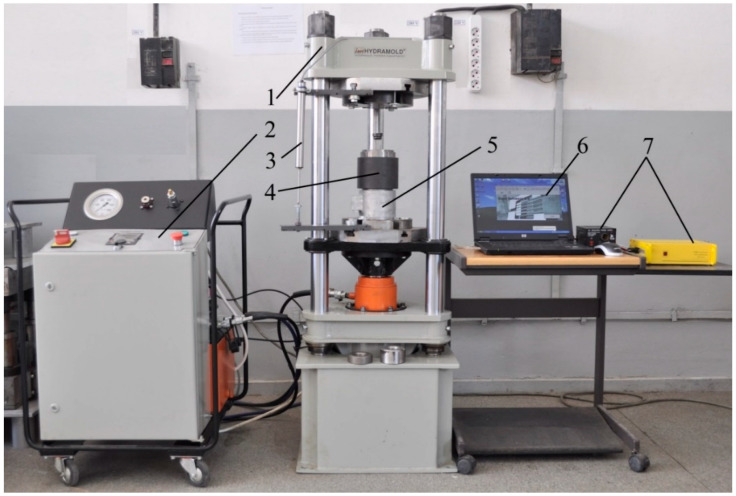
General view of the setup for determining the deformation force. 1—press; 2—hydraulic unit; 3—displacement transducer; 4—deformation device; 5—load cell; 6—notebook; 7—Traveller 1 system.

Biomechanical testing was conducted using a 750 kN hydraulic press (Hidramold 750 kN, Fabricated by Hidramold, Iasi, Romania) at the Materials Engineering and Industrial Safety Laboratory of the “Gheorghe Asachi” Technical University of Iași. The press was equipped with a 1000 kN force transducer and a 0–100 mm displacement transducer to capture real-time data on force and displacement during testing (Figure 7).

All the transducers were calibrated prior to testing to ensure accurate force and displacement measurements. The data acquisition system, Master Unit System Traveller 1 (MUT-1), was used to capture data through eight strain gauge amplification channels. The system was capable of sampling up to 100,000 data points per second. The data were recorded using E.S.A.M (software version 3.0).

### 2.5. Experimental Procedure

Two types of mechanical tests were conducted to evaluate the biomechanical performance of the K-wire configurations:

Axial Compression Test: Each specimen was mounted vertically in the hydraulic press, with the distal end of the bone positioned against the loading plate. A compressive force was applied at a constant rate of 0.4 mm/s until structural failure occurred. Failure was defined as a significant drop in the load-bearing capacity. The maximum force (in Newtons) and corresponding displacement (in millimeters) were recorded. This test simulated axial loading conditions similar to those experienced by the distal radius during weight-bearing activities.

Flexion Test with dorsal displacement: For the flexion test, using the same machine, each specimen was mounted horizontally in a custom fixture designed to simulate wrist flexion under a dorsally applied load. A three-point bending setup was used, with the load applied 5 mm proximal to the fracture site. The force was applied at a constant rate until failure occurred, defined by a significant drop in the load-bearing capacity. The peak load and displacement at failure were recorded. This test simulated the forces experienced during a fall onto an outstretched hand, a common mechanism of distal radius fractures.

### 2.6. Specimen Handling

All the specimens were tested as soon as possible after preparation to ensure the reliability of the results. No freezing or preservation methods were used, as immediate testing ensured that bone properties remained consistent across all the specimens, and avoided downsides like degradation over time [22].

### 2.7. Data Analysis

Data from the mechanical tests were analyzed using the SPSS software (version 27). Paired *t*-tests were performed to compare the performance of different K-wire configurations and sizes. A significance threshold of *p* < 0.05 was applied. In addition to the mean and standard deviation (SD), 95% confidence intervals (CIs) were calculated for all the key parameters, including axial stiffness, flexion stiffness, and load at break. CI was used to estimate the precision of the results and provide additional insights into the reliability of the findings.

## 3. Results

This study aimed to evaluate the biomechanical performance of three-wire Kirschner (K) wire configurations, comparing the intra-focal (Ka) and interfragmentary (IF) techniques in axial stiffness and load at break. Previous studies have shown that three-wire configurations outperform two-wire configurations in terms of stiffness and load capacity, making the three-wire setup the focus of this study. The results show that the interfragmentary configuration provided better overall mechanical performance across all the tests, and the use of 2.0 mm K-wires resulted in better outcomes compared to the 1.6 mm K-wires.

### 3.1. Axial Stiffness in Compression

Table 1 presents the results for axial stiffness under compression. The interfragmentary configuration demonstrated significantly greater stiffness than the intra-focal configuration for both the 1.6 mm and 2.0 mm K-wires. Specifically, for the 1.6 mm K-wires, the interfragmentary configuration showed an axial stiffness of 11.25 MPa (95% CI: 10.00 to 12.50) compared to 3.91 MPa (95% CI: 3.48 to 4.34) for the intra-focal configuration (*p* = 0.034). Similarly, for the 2.0 mm K-wires, the interfragmentary technique demonstrated 13.28 MPa (95% CI: 11.81 to 14.75) compared to 9.38 MPa (95% CI: 8.34 to 10.42) for the intra-focal configuration (*p* = 0.045).

**Figure 8:**
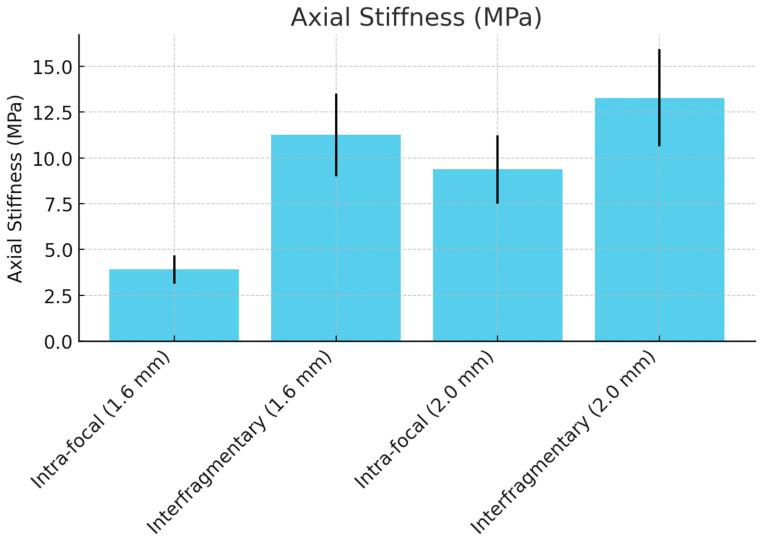
Axial stiffness comparison between intra-focal and interfragmentary K-wire techniques.

The comparison between wire sizes showed that the interfragmentary configuration benefitted more from increasing the wire size, with a significant increase from 1.6 mm to 2.0 mm K-wires (*p* = 0.018 for IF, *p* = 0.015 for Ka), as shown in (Figure 8).

### 3.2. Flexion Stiffness with Dorsally Displacing Force

Table 2 summarizes the flexion stiffness results. For the 1.6 mm K-wires, the interfragmentary configuration exhibited slightly higher stiffness (67.75 MPa, 95% CI: 60.25 to 75.25) compared to the intra-focal configuration (64.48 MPa, 95% CI: 57.34 to 71.62), although the difference was not statistically significant (*p* = 0.056). However, for 2.0 mm K-wires, the interfragmentary configuration (103.40 MPa, 95% CI: 91.95 to 114.85) demonstrated significantly greater stiffness compared to the intra-focal configuration (65.26 MPa, 95% CI: 58.03 to 72.49) (*p* = 0.028).

**Figure 9:**
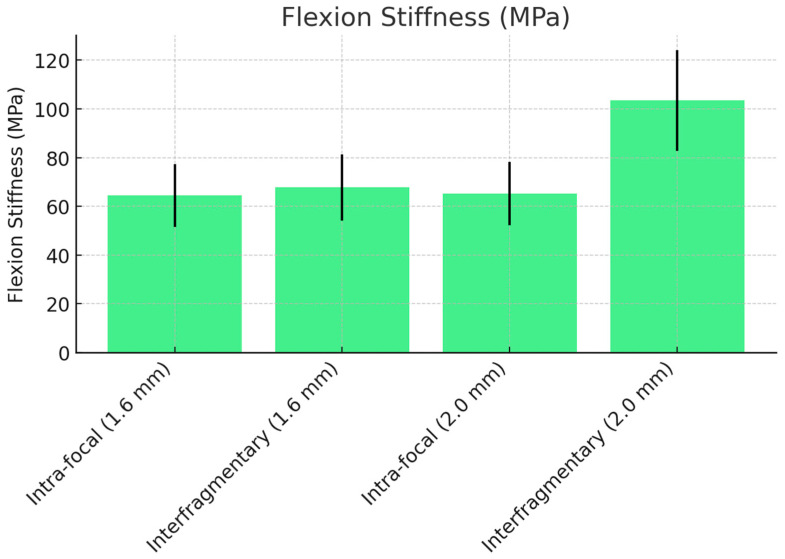
Flexion stiffness comparison between intra-focal and interfragmentary K-wire techniques.

In comparing wire sizes, flexion stiffness increased significantly with 2.0 mm K-wires in the interfragmentary configuration (*p* = 0.022), while the increase in the intra-focal configuration was not statistically significant (*p* = 0.074), as shown in (Figure 9).

### 3.3. Axial Load at Break

Table 3 presents the mean axial load at break. For the 1.6 mm K-wires, the interfragmentary configuration demonstrated a significantly higher load at break (2920 N, 95% CI: 2596.59 to 3243.41) compared to the intra-focal configuration (1944 N, 95% CI: 1728.69 to 2159.31) (*p* = 0.041). Similarly, with the 2.0 mm K-wires, the interfragmentary configuration (3070 N, 95% CI: 2729.98 to 3410.02) outperformed the intra-focal configuration (2230 N, 95% CI: 1983.01 to 2476.99) with a statistically significant difference (*p* = 0.049).

**Figure 10:**
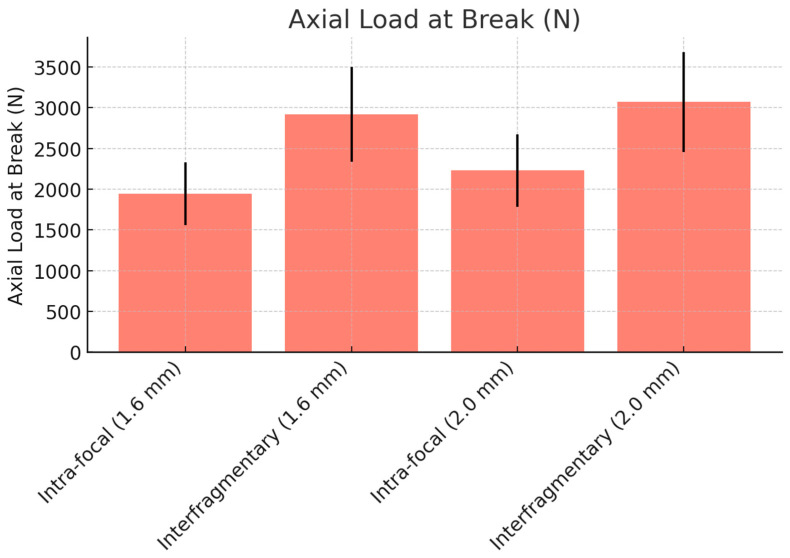
Axial load at break comparison between intra-focal and interfragmentary K-wire techniques.

The comparison of wire sizes showed a significant improvement with 2.0 mm K-wires in both configurations (*p* = 0.012 for IF, *p* = 0.034 for Ka), as shown in (Figure 10).

### 3.4. Flexion Load at Break

The results for the load at break during flexion are presented in Table 4. For the 1.6 mm K-wires, the interfragmentary configuration withstood significantly higher loads (815 N, 95% CI: 652 to 978 N) compared to the intra-focal configuration (320 N, 95% CI: 256 to 384 N) (*p* = 0.032). Similarly, for the 2.0 mm K-wires, the interfragmentary configuration (920 N, 95% CI: 736 to 1104 N) exhibited greater load capacity compared to the intra-focal configuration (680 N, 95% CI: 544 to 816 N) (*p* = 0.027).

## 4. Discussion

### 4.1. Significance Summary

The findings from this study consistently demonstrate the biomechanical superiority of the interfragmentary K-wire configuration over the intra-focal technique in terms of both axial stiffness and load-bearing capacity. Across all the tests, the use of the 2.0 mm K-wires yielded significantly better outcomes compared to the 1.6 mm wires, especially in the interfragmentary setup. These results are supported by narrow confidence intervals, underscoring the robustness and reliability of the findings.

Our results are in line with prior research, which has suggested that thicker K-wires and the use of multi-wire configurations, particularly the three-wire setup, provide greater mechanical stability for distal radius fracture fixation [21,23,24]. This study expands on earlier work by focusing on the comparative effectiveness of wire thickness, showing that the interfragmentary configuration with the 2.0 mm K-wires offers significant mechanical advantages over the intra-focal technique.

### 4.2. Comparison with Existing Literature

The biomechanical advantages of the interfragmentary technique observed in this study are consistent with findings from another study [19], which demonstrated that wire configuration and wire thickness are crucial factors influencing fracture stability. Previous studies have also shown that multi-wire configurations, such as the three-wire interfragmentary method, provide enhanced fracture stability by increasing the number of bony contact points, which helps to distribute loads more effectively [12,14,21,24].

Our study further contributes to the understanding of how wire diameter impacts fracture fixation, with thicker wires (2.0 mm) proving more effective at resisting axial loads and enhancing flexion stiffness. Thicker K-wires provide greater resistance to postoperative displacement in extra-articular comminuted fractures [25,26].

However, previous research has indicated that K-wire fixation may be less effective in osteoporotic or elderly patients due to bone fragility [14,27,28]. While our findings suggest that thicker K-wires improve biomechanical performance, more studies are needed to verify this in osteoporotic bone models [24,28]. Additionally, while our study supports the use of turkey tarsometatarsus bones as an adequate analog for human distal radius bones [12,15,29], differences in bone quality between species, particularly in terms of density and mechanical behavior, may limit the clinical applicability of these results—especially for osteoporotic patients [18,24,28]. Future studies should focus on human bone models, particularly in elderly populations with varying bone densities [28,29].

### 4.3. Clinical Implications

Clinically, the superior biomechanical performance of the interfragmentary technique suggests that it should be the preferred method for fixing distal radius fractures, particularly in cases requiring enhanced stability, such as extra-articular comminuted or high-energy trauma fractures. The higher load-bearing capacity and stiffness provided by the 2.0 mm K-wires may reduce the risk of postoperative complications, such as fracture displacement or malunion, thereby potentially improving long-term patient outcomes [19,27]. This is further supported by the theory that interfragmentary wires provide enhanced stiffness due to having two bony insertion points per wire, in contrast to the intra-focal technique, where each wire has only one insertion point, resulting in superior mechanical stability in the interfragmentary setup [12].

Moreover, K-wire fixation is efficient and cost-effective, as demonstrated in various studies [25,26], with studies indicating no significant difference in functional outcomes between the two techniques for dorsally displaced distal radius fractures [30]. K-wires are quicker to use and more accessible, particularly in resource-limited settings, making the interfragmentary technique a practical option for maintaining fracture stability and reduction.

### 4.4. Limitations

However, while our study demonstrates the biomechanical superiority of the interfragmentary technique, further clinical trials are necessary to determine how these biomechanical advantages affect functional recovery and outcomes [30,31]. Postoperative rehabilitation, which plays a key role in patient recovery, was not addressed in this study, and future research should explore how different rehabilitation protocols could complement the biomechanical benefits observed [32,33,34].

Despite the promising results, this study has several limitations. First, while the turkey tarsometatarsus model has been shown to be a suitable analog for human distal radius bones [12,15,29], it may not fully replicate the biomechanical behavior of human bones, particularly in osteoporotic or elderly patients. Bone quality in human patients can vary significantly due to factors such as age and osteoporosis, which may influence the mechanical properties of fracture fixation [14,24,30]. Additionally, although we aimed to preserve as much soft tissue as possible within the turkey bone models, the lack of soft tissue structures, such as ligaments, tendons, and muscles, may limit the ability to replicate the complete biomechanical environment present in human distal radius fractures, where soft tissues play a critical role in fracture stability and healing. Future research should incorporate more sophisticated models that account for these soft tissue factors, as well as osteoporotic bone models, or conduct clinical trials on human subjects to better assess the applicability of these findings in real-world scenarios.

Additionally, while we observed significant biomechanical advantages of the interfragmentary configuration, these results are based solely on mechanical testing. To fully translate these biomechanical benefits into clinical practice, further studies are required. Specifically, long-term clinical trials involving patient-reported outcome measures (PROMs) would provide valuable insights into whether the increased biomechanical stability translates into better recovery, function, and patient satisfaction [14,31].

### 4.5. Future Directions

Future research should expand on these findings by examining how different wire configurations and thicknesses affect outcomes in osteoporotic bone models, as noted in prior studies [18,29,30]. While our results are consistent with much of the existing literature, further exploration is necessary to address the conflicting data on the best fixation techniques, especially in the context of osteoporotic or complex fractures [30,35]. A more in-depth look at combining K-wires with other methods, like volar plates or external fixators, could offer a clearer picture of the advantages and limitations of these approaches for comminuted distal radius fractures [6,28,33]. Additionally, future studies on postoperative rehabilitation could shed light on whether early mobilization enhances recovery after K-wire fixation [32]. This broader approach will help refine the current treatment practices and fill important gaps in the literature.

## 5. Conclusions

This study provides a comparative biomechanical evaluation of two commonly used K-wire techniques—intra-focal and interfragmentary—for stabilizing dorsally displaced distal radius fractures. The findings clearly demonstrate that the interfragmentary technique, especially when using 2.0 mm K-wires, delivers significantly better performance in terms of stiffness and load-bearing capacity. This suggests that wire diameter and configuration are crucial factors for optimizing fracture stability and minimizing complications, such as malunion or fracture displacement.

The enhanced mechanical stability offered by the interfragmentary technique is likely due to the increased contact points within the bone, allowing for a better distribution of forces. Clinically, this stability could reduce postoperative issues like the loss of fracture alignment, improving long-term functional outcomes. Additionally, the interfragmentary method presents a cost-effective alternative to more expensive surgical options, such as volar locking plates, making it especially appealing in resource-constrained settings.

However, while the biomechanical results are promising, further studies are needed to confirm how these findings translate into clinical practice, especially regarding patient outcomes. Investigating the effects of rehabilitation protocols could also be useful in determining whether early mobilization is feasible with this technique, potentially enhancing recovery.

In conclusion, this study underscores the advantages of the interfragmentary K-wire configuration, particularly with 2.0 mm wires, in providing superior mechanical stability for distal radius fracture fixation. These results can help inform future surgical practices and contribute to improving patient care through more effective and accessible fracture management techniques.

## Figures and Tables

**Figure 11:**
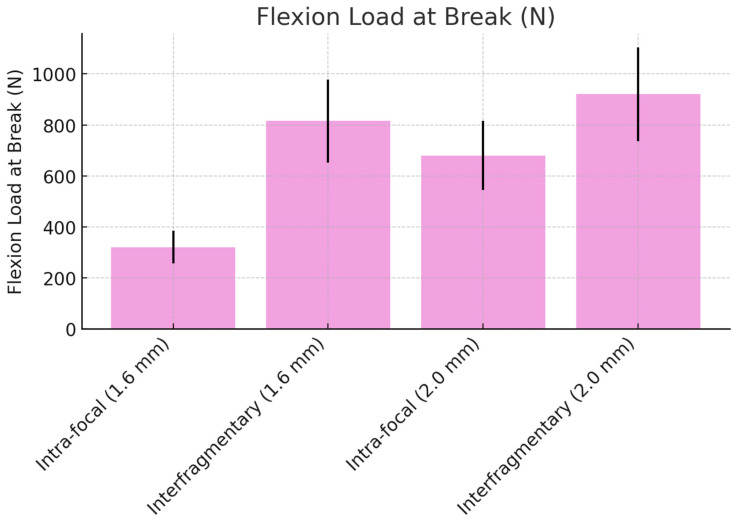
Flexion load at break comparison between intra-focal and interfragmentary K-wire techniques.

**Table 1:** Mean stiffness in axial compression (MPa).

Axial Stiffness (MPa)	Intra-Focal (Ka) Mean ± SD (95% CI)	Interfragmentary (IF) Mean ± SD (95% CI)	Ka vs. IF *p*-Value
1.6 mm K-wire	3.906 ± 0.78 (3.48 to 4.34)	11.250 ± 2.25 (10.00 to 12.50)	0.034
2.0 mm K-wire	9.375 ± 1.87 (8.34 to 10.42)	13.281 ± 2.66 (11.81 to 14.75)	0.045
1.6 mm vs. 2.0 mm K-wire *p*-value	0.015	0.018	

**Table 2:** Mean stiffness in flexion with a dorsally displacing force (MPa).

Flexion Stiffness (MPa)	Intra-Focal (Ka) Mean ± SD (95% CI)	Interfragmentary (IF) Mean ± SD (95% CI)	Ka vs. IF *p*-Value
1.6 mm K-wire	64.480 ± 12.90 (57.34 to 71.62)	67.752 ± 13.55 (60.25 to 75.25)	0.056
2.0 mm K-wire	65.259 ± 13.05 (58.03 to 72.49)	103.404 ± 20.68 (91.95 to 114.85)	0.028
1.6 mm vs. 2.0 mm K-wire *p*-value	0.074	0.022	

**Table 3:** Mean load at break in axial compression (N).

Flexion Stiffness (MPa)	Intra-Focal (Ka) Mean ± SD (95% CI)	Interfragmentary (IF) Mean ± SD (95% CI)	Ka vs. IF *p*-Value
1.6 mm K-wire	1944 ± 388.8 (1728.69 to 2159.31)	2920 ± 584 (2596.59 to 3243.41)	0.041
2.0 mm K-wire	2230 ± 446 (1983.01 to 2476.99)	3070 ± 614 (2729.98 to 3410.02)	0.049
1.6 mm vs. 2.0 mm K-wire *p*-value	0.034	0.012	

**Table 4:** Mean load at break in flexion with a dorsally displacing force (N). The 0 mm K-wires in the interfragmentary configuration (*p* = 0.015), while the increase in the intra-focal configuration was not statistically significant (*p* = 0.084). These results are illustrated in (Figure 11).

Flexion Stiffness (MPa)	Intra-Focal (Ka) Mean ± SD (95% CI)	Interfragmentary (IF) Mean ± SD (95% CI)	Ka vs. IF *p*-Value
1.6 mm K-wire	320 ± 64 (256 to 384)	815 ± 163 (652 to 978)	0.032
2.0 mm K-wire	680 ± 136 (544 to 816)	920 ± 184 (736 to 1104)	0.027
1.6 mm vs. 2.0 mm K-wire *p*-value	0.084	0.015	

## Data Availability

The original contributions presented in this study are included in the article. Further inquiries can be directed to the corresponding authors.
